# Classifying the Perceptual Interpretations of a Bistable Image Using EEG and Artificial Neural Networks

**DOI:** 10.3389/fnins.2017.00674

**Published:** 2017-12-04

**Authors:** Alexander E. Hramov, Vladimir A. Maksimenko, Svetlana V. Pchelintseva, Anastasiya E. Runnova, Vadim V. Grubov, Vyacheslav Yu. Musatov, Maksim O. Zhuravlev, Alexey A. Koronovskii, Alexander N. Pisarchik

**Affiliations:** ^1^REC “Artificial Intelligence Systems and Neurotechnology”, Yuri Gagarin State Technical University of Saratov, Saratov, Russia; ^2^Faculty of Nonlinear Processes, Saratov State University, Saratov, Russia; ^3^Center for Biomedical Technology, Technical University of Madrid, Madrid, Spain

**Keywords:** brain, ambiguous image, multistability, EEG, artificial neuronal network, brain states recognition

## Abstract

In order to classify different human brain states related to visual perception of ambiguous images, we use an artificial neural network (ANN) to analyze multichannel EEG. The classifier built on the basis of a multilayer perceptron achieves up to 95% accuracy in classifying EEG patterns corresponding to two different interpretations of the Necker cube. The important feature of our classifier is that trained on one subject it can be used for the classification of EEG traces of other subjects. This result suggests the existence of common features in the EEG structure associated with distinct interpretations of bistable objects. We firmly believe that the significance of our results is not limited to visual perception of the Necker cube images; the proposed experimental approach and developed computational technique based on ANN can also be applied to study and classify different brain states using neurophysiological data recordings. This may give new directions for future research in the field of cognitive and pathological brain activity, and for the development of brain-computer interfaces.

## 1. Introduction

The brain is likely the most convoluted and enigmatic research object, attracting the burning interest of the broad scientific community in diverse areas of science and technology, including neurophysiology, medicine, engineering, physics, and mathematics (Wolf, 2005; Bick and Rabinovich, 2009; Chavez et al., 2010; van Luijtelaar et al., 2011; Bear et al., 2015; Hramov et al., 2015). One of the important problems in the field of brain research is the cognitive brain function during visual perception. For a long time, this problem has attracted a lot of attention of various researchers, especially in connection with such important tasks as object recognition (Martin, 2007; Müler et al., 2008; Simanova et al., 2010; Isik et al., 2014) and decision making (Heekeren et al., 2008; Wang, 2008, 2012). Nowadays, these tasks are of great practical importance for the development of novel communication, computer technologies, and robotics.

Visual perception, object recognition, and decision-making processes in human brain are often studied with the help of *ambiguous* visual stimuli, also known as *bistable* or *multistable*) images (Schwartz et al., 2012; Cao et al., 2014). Different interpretations of a bistable image (Leopold and Logothetis, 1999; Blake and Logothetis, 2002; Pisarchik et al., 2014, 2015) are tightly connected with the problem of categorical perception in humans (Etcoff and Magee, 1992) and non-human primates (Freedman et al., 2001; Liu and Jagadeesh, 2008). Among popular examples of bistable images are Rubin vase, Mach bands, Rorschach test, Boring's old woman/young woman illusion, and Necker cube. For a long time, ambiguous images have been research objects for psychologists (Leopold and Logothetis, 1999; Sterzer et al., 2009). Recently, such images awoke a growing interest of physicists and mathematicians to study stochastic perception models using noise to convert multistable systems into metastable ones (Pisarchik et al., 2014, 2015; Runnova et al., 2016; Bashkirtseva and Ryashko, 2017). Although the underlying mechanism of image recognition is not yet well understood, the metastable visual perception is known to engage a distributed network of occipital, parietal, and frontal cortical areas (Tong et al., 2006; Sterzer et al., 2009).

When a subject observes an ambiguous object for a sufficient length of time, he shows individual features of alternative switching between different percepts, e.g., the Rubin vase is alternately perceived as two faces and a vase (Leopold and Logothetis, 1999); the Necker cube can be interpreted as a left-oriented or a right-oriented cube (Borsellino et al., 1972). According to existing hypothesis, the switches in perception are caused by stochastic processes in the brain neural network due to spontaneous neural activity: random generation of membrane potentials and random synaptic connections (Merk and Schnakenberg, 2002; Moreno-Bote et al., 2007; Gigante et al., 2009; Huguet et al., 2014). These random neural background activity plays a crucial role in the interpretation of ambiguous images and other decision-making tasks. Following this hypothesis, perception of ambiguous objects can be described by simple stochastic models, like Weiner dynamics (Aks and Sprott, 2003; Ratcliff and Smith, 2004; Heekeren et al., 2008; Wang, 2012; Pearson et al., 2014; Runnova et al., 2016). It is clear that the brain states description and classification during the decision-making process open wide perspectives for a deeper understanding of the mechanisms responsible for visual spatial perception in the human brain with a strong stochastic component, and create also the possibility for perception control (Pisarchik et al., 2015).

One of the most appropriate techniques for studying brain states is based on the analysis of multichannel electro encephalographic (EEG) signals (Cooper et al., 1980; Tatum, 2014). In the context of bistable perception, the analysis of the EEG data allows one to reveal specific features of the perceptive process. In particular, Kornmeier et al. (2011) discovered two types of EEG signatures, stimulus-related (low-level) and percept-related (high-level) during perception of the Necker cube. The percept-related features associated with the Necker cube reversals were found in gamma (Strüber et al., 2001) and delta (Mathes et al., 2006) frequency bands. Thus, different brain states manifest themselves as specific oscillatory patterns in EEG signals characterized by a particular time-frequency structure. This gives us the possibility to detect and classify the brain states by processing the EEG data (Donner et al., 2009).

Among various approaches proposed for the classification of oscillatory patterns observed in the EEG recordings (Garrett et al., 2003; Dias et al., 2007; Siuly et al., 2016), some are worth mentioning such as discriminant analysis methods (which were very popular in the 1960s) (Niedermeyer and Lopes da Silva, 2005; Hasan et al., 2015), independent component analysis (Makeig et al., 1996; Ungureanu et al., 2004; Hobson and Hillebrand, 2006) (often used for finding and eliminating biased artifacts in EEG signals; Jung et al., 2000), short-time Fourier transform (Gotman et al., 1973), and wavelet-based methods (Hramov et al., 2015), including techniques of adaptive mother wavelets (Sitnikova et al., 2009; Nazimov et al., 2013) and methods based on estimation of event-related synchronization/desynchronization (Morash et al., 2008). Nowadays, another classification technique known as *artificial neural network* (ANN) (Bishop, 2006; Haykin, 2008) is widely used in computer science, biophysics, deep learning, econometrics, etc. (Bishop, 1996; Goodfellow et al., 2016; Zhou et al., 2017). This method inspired by biological interconnected neurons is based on nonlinear models of neural units (artificial neurons). The ANNs can be either hardware-based (neurons represented by physical components) or software-based (computer models), and can use a variety of topologies and learning algorithms. Hardware ANNs are more accurate in mimicking the performance of real neural networks and have a higher performance speed than software-based ANNs. Due to these features hardware ANNs should be better for real-time implementation, but they heavily rely on a specific hardware configuration. In contrast, software ANNs have a more simple and therefore a more flexible structure and can be easily implemented in practice (Baptista et al., 2013).

Many types of architectures of software-based ANNs were developed to solve different relevant tasks. In particular, convolutional neural networks (CNN) were applied for image recognition and also for pattern recognition in EEG signals (Hajinoroozi et al., 2016). CNNs are very efficient in revealing specific features in unstructured data, like images, audio and video. However, in spite of their excellent properties in pattern recognition and classification, CNNs require a relatively large number of varied parameters for each task and most of them can only be tuned empirically. Therefore, in such a specific task as the classification of undetermined types of EEG patterns, it is more convenient to use simpler and hence more flexible ANNs, such as multilayer perceptron (MLP) (Haselsteiner and Pfutscheller, 2000).

In this paper, we propose using the MLP for the classification of the human EEG recorded during visual perception of ambiguous images, the classical example being the Necker cube, named after the Swiss mathematician and physicist Louis Albert Necker (1730–1804) (Necker, 1832). This cube represents a contour image with reversible perspective corresponding to the parallel projection of nodes and edges of the cube onto the plane, disregarding the rules of perspective. During the perception of this figure, the person observes spontaneous flips, i.e., one volumetric projection is replaced by another. An important advantage of the Necker cube over many other ambiguous images, is that its ambiguity can be digitalized and controlled by varying the cube parameters, such as the angle of displacement between planes, thickness of outlines, filling of sides with color or shade (Taeed et al., 1988). We prove that the ANN technique enables us to distinguish with high precision between particular EEG features caused by different cube orientations. Moreover, when we apply ANN in a cross-subject mode, we discover the existence of universal patterns in EEG, common for all subjects. Unlike previous studies on the use of artificial intelligence for classification of EEG traces (Ma et al., 2017; Quitadamo et al., 2017), where the maximal precision was achieved by subject-oriented adjustment, our approach, along with high-quality classification, gives some common information about the brain's response to the bistable stimuli. Here, we discuss the possibility of the ANN to reveal cognitive brain properties of visual perception and compare the ANN with other approaches, often used for such purposes. Along with ANN, we also apply event related potentials (ERP) and wavelet-based approaches to detect features of the brain states, associated with different interpretations of the Necker cube. We demonstrate the advantages of the ANN in revealing more pronounced differences in brain states among all subjects. Finally, we expect our results to be useful in interdisciplinary fundamental research and brain-computer interfaces.

The structure of the paper is as follows. In section 2 we describe materials and methods used in our neurophysiological experiments on EEG recordings, as well as subjects and experimental procedure. The results of data processing and classification of brain states using ANN during visual perception of ambiguous images, are given in section 3 and discussed in section 4. Finally, the main conclusion is given in section 5.

## 2. Materials and methods

### 2.1. Experimental setup and subjects

**Figure 1:**
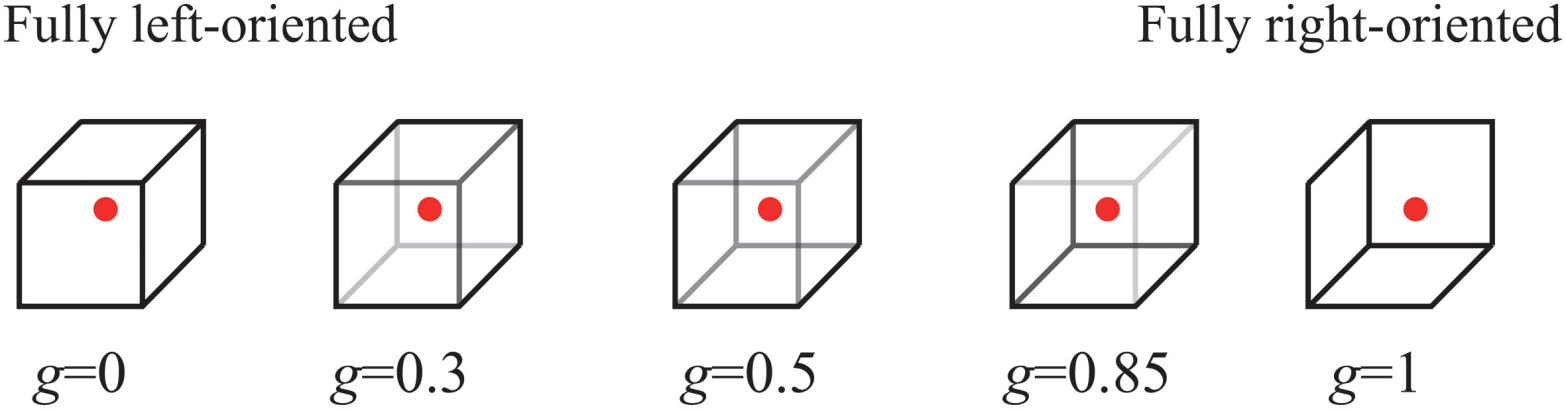
Examples of distinct Necker cube images with different wireframe contrasts characterized by control parameter *g*. The left-hand image with *g* = 0 corresponds to the fully left-oriented cube, while the right-hand image with *g* = 1 to the fully right-oriented cube. Each subject was instructed to fix his/her sight at the central red dot.

Subjects were facing a display screen on which ambiguous images were displayed as visual stimulus (see Figure 1). As an ambiguous image, we used the Necker cube (Necker, 1832), a flat 2D-image which due to optical illusion looks like a cube with transparent faces and visible ribs. Visual bistability consists in the fact that this 3D-object can be treated as oriented in two different ways, especially if different ribs of the Necker cube are drawn with different intensity. Specifically, the contrast of the three middle lines centered in the left middle corner, *g* ∈ [0, 1], was used as a control parameter of the displayed images. The boundary values *g* = 1 and *g* = 0 correspond, respectively, to 0 (black) and 255 (white) pixels' luminance of the middle lines, using the 8-bit grayscale palette for visual stimulus presentation. Therefore, we can define a contrast parameter as *g* = *b*/255, where *b* is the brightness level of the middle lines in the used 8-bit grayscale palette. The contrast of three middle lines centered in the right middle corner was set to (1 − *g*), and the normalized contrast of the six visible outer cube edges was fixed to 1. The Necker cube images with ribes of different intensities *g* were created using a standard graphics software.

The multi-channel EEG was recorded at a 250-Hz sampling rate with *P* = 19 electrodes and two reference electrodes placed at standard positions of the 10–20 international system (Niedermeyer and da Silva, 2004). To register the EEG data we used cup adhesive Ag/AgCl electrodes placed on the “10–20” paste. Immediately before the experiment started, we performed all necessary procedures to increase the conductivity of the skin and reduce its resistance using abrasive “NuPrep” gel. The impedances were monitored after the electrodes were installed, and measured during the experiments. Usually, the impedance values varied within the 2–5 kΩ interval. The ground electrode N was located in front of the head at the Fpz electrode location. The EEG signals were filtered by a band-pass filter with cut-off points at 1 Hz (HP) and 100 Hz (LP) and a 50-Hz Notch filter. The electroencephalograph “Encephalan–EEGR–19/26” (Medicom MTD company, Taganrog, Russian Federation) with multiple EEG channels and two-button input device (keypad), was used for amplification and analog-to-digital conversion of the EEG signals. Electroencephalograph “Encephalan–EEG–19/26” possesses the registration certificate of the Federal Service for Supervision in Health Care No. FCP 2007/00124 of 07.11.2014 and the European Certificate CE 538571 of the British Standards Institute (BSI). Preliminary signal processing was provided by the original software for EEG registration artifact suppression. To exclude artifacts associated with eye movement we used the electrooculogram (EOG) signals from two pairs of electrodes placed on the eye socket, above (or below) and at eye level. The EOG signals were filtered with the same band-pass filter and registered by the “Encephalan–EEGR–19/26” equipment.

Machine learning algorithms were implemented with MATLAB. To demonstrate a grayscale stimulus, we used a 24″BenQ LCD monitor with a spatial resolution of 1, 920 × 1, 080 pixels and a refresh rate of 60 Hz. For the presentation of visual stimuli we used the system “ABC–stimulus” included in the Medicom MTD software for electroencephalograph “Encephalan–EEGR–19/26”. The “ABC–stimulus” software provided highly precise time synchronization of the EEG recording and of the stimulus presentation based on a special software and an additional video sensor attached to the monitor. Each Necker cube image drawn by black and gray lines was located at the center of the computer screen on a white background. A red dot drawn at the center of the Necker cube was used to attract the attention of subjects and prevent possible perception shifts due to eye movements while observing the image. The subjects were located at a distance of 70–80 cm from the monitor with a visual angle of approximately 0.25 rad. The Necker cube size on the monitor was 14.2 cm.

The experimental studies were performed in accordance to Ethical Standards (2000) and approved by the local research Ethics Committee of the Yuri Gagarin State Technical University of Saratov. Twelve healthy subjects from a group of unpaid volunteers, male and female, between the ages of 20 and 45 with normal or corrected-to-normal visual acuity participated in the experiments. Written informed consent was obtained from all participants.

### 2.2. Experimental design

Every subject completed a single recording session to avoid possible adaptation and brain adjustment when solving the task. During the experiment, seven Necker cube images (*M* = 7) with different wireframe contrasts, i.e., with seven different values of the control parameter *g*_*i*_ = 0.15, 0.3, 0.4, 0.5, 0.6, 0.7, 0.85, were randomly presented to each subject. All participants were aware about the two possible cube interpretations and able to see both. When observing the Necker cube, the mean duration of a visual percept is known to vary from one second to several minutes for different subjects and stimulus conditions (Pastukhov et al., 2013), whereas the mean response time is rather consistent and varies only by a few hundred ms among all of subjects and stimulus conditions (Carpenter, 2012). In literature, the experimentally measured typical duration of one of the percepts of the Necker cube was found to be approximately 1 s (Merk and Schnakenberg, 2002).

**Figure 2:**
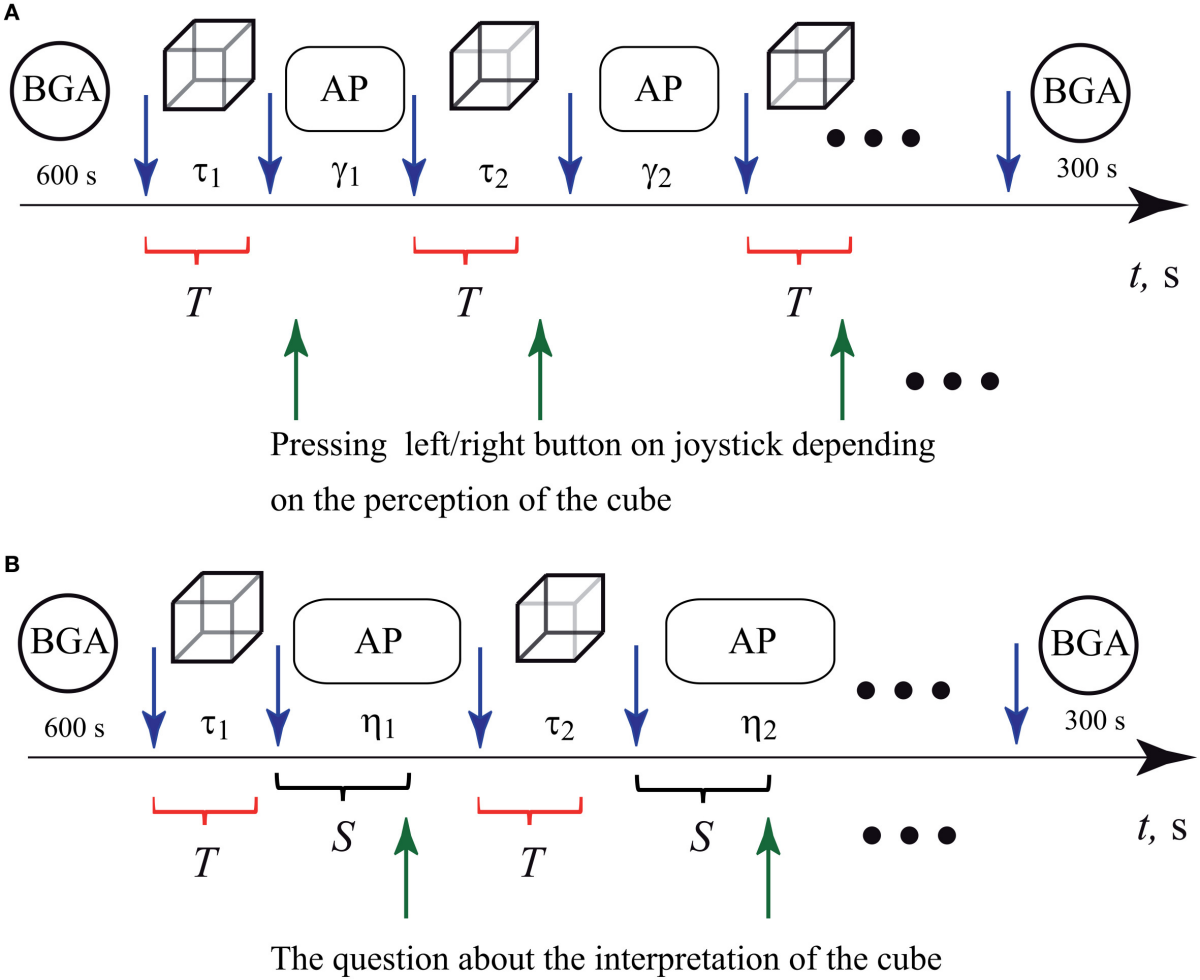
Schematic representation of two sets of experiments. Interpretation of the Necker cube as left- or right-oriented by **(A)** pressing the corresponding button on the keypad and **(B)** answering experimenter's questions concerning the cube orientation. The blue upper arrows before and after every cube presentation bound the epochs of durations τ_1_, τ_2_, …τ_*N*_ (τ = 0.8 − 1.3 s, *N* = 400) when the cube with randomly chosen control parameter *g*_*j*_ (*j* = 1, …*M*, *M* = 7) was presented to the observer. The circles at the beginning and at the end of both sets of the experiments indicate time intervals during which the background EEG activity (BGA) was recorded. The ovals indicate ISIs γ_1_, γ_2_, …γ_*N*_ (γ = 2 − 3 s, *N* = 400) in the first and η_1_, η_2_, …γ_*N*_ (η = 5 − 7 s, *N* = 200) in the second set of experiments, during which abstract pictures (AP) were presented. In **(A)**, the green lower arrows indicate the moments when the subject pressed either left or right button while interpreting the Necker cube as left- or right-oriented. In **(B)**, the green lower arrows indicate the moments when the experimenter asked the subject about her/his interpretation of the cube orientation. The time interval between the end of the cube presentation and the moment when the experimenter asked the question was equal to *S* = 3 s. The red brackets below the time axis indicate time intervals *T* of the EEG trials taken for the analysis by means of artificial neuronal networks.

We carried out two sets of experiments. In the first set, all participants were instructed to press either a left or a right key on the two-button keypad according to their first visual impression on the cube orientation (left-oriented or right-oriented), as shown in Figure 2A. In the second set shown Figure 2B, the subjects did not need to press the buttons. In order to exclude the effect of motor reaction after the image presentation, the experimenter asked the participant to interpret the cube orientation to be either “left” or “right” and then voice her/his interpretation.

It is known that when visual stimuli are subsequently presented to the observer, the effect of stabilization of visual perception takes place (Leopold et al., 2002). This effect consists in persistent visual perception between subsequent presentations of images. Even though several model-based approaches (Wilson, 2007) have been proposed to explain this phenomenon, the truth is that the underlying mechanism of the stabilization effect is not yet well understood. To diminish the stabilization effect, we diverted the subject's attention by exhibiting abstract pictures for about γ = 2 − 3 s in the first set and η = 5 − 7 s in the second set, between subsequent demonstrations of the Necker cube images, in order to guarantee as much as possible the independence of two consecutive Necker cube images. Also, we sought to fix the first impression and avoid switches between two possible percepts during the image demonstration by imposing a short exhibition time τ_*i*_ = 0.8 − 1.3 s.

It should be noted that the duration of the stimuli presentations, τ_*i*_, as well as inter-stimulus intervals (ISIs), γ_*i*_ (for button response) and η_*i*_ (for voice response), were randomly chosen from the defined above time intervals. The ISIs were chosen to be sufficiently large to diminish the influence of the stabilization effect. The ISIs for voice response, η_*i*_, were chosen to be larger than γ_*i*_ in order to have enough time to ask the subject about his/her interpretation of the cube orientation.

The schematic representations of two experimental designs are given in Figure 2. In both experiments we started with the recording of the background EEG activity when the subject was in a relaxed state (BGA in Figure 2). The duration of the background recording was 600 s. We finished the experiments by recording again the background EEG activity (BGA) during 300 s.

#### First set of experiments (with key pressing)

The following protocol was used at each run of the first set of experiments, i.e., when a key was pressed.

After recording the background EEG activity, we began the main part of the experiment demonstrating the Necker cubes with different values of the control parameter *g*. This stage consisted of three steps.

1. The visual stimulus (the Necker cube with randomly chosen contrast parameter *g*_*j*_) was displayed on the screen during time interval τ_*i*_ randomly chosen between 0.8 and 1.3 s.
2. After observing the Necker cube on the screen, the subject analyzed its position and pressed either left or right button on the keypad depending on his/her first visual impression to indicate the cube orientation. We did not regulate the method of keystrokes; the subjects usually pressed the left or right key by index fingers or thumbs of the left or right hand, respectively.
3. Between subsequent demonstrations of the Necker cubes, abstract pictures (AP) were exhibited during time γ_*i*_ randomly chosen in the 2–3 s interval to divert attention and make the perception of the next image independent of the previous one.
4. Steps (1–3) were repeated *N* = 400 times.

During the data acquisition for each subject lasted about 40 min, 400 Necker cube images were presented. The time markers of the cube and abstract image presentations (start and finish), type of image (parameter *g*), the moments when the subject pressed a button on the keypad (left or right), as well as the selection of the type of perception (left- or right-oriented cube) were automatically recorded during the experiment and saved in a special log-file for further analysis.

#### Second set of experiments (with voice response)

The second set of experiments was designed as follows. After recording the background EEG activity, we entered to the main part of the experiment demonstrating the Necker cubes with different values of the control parameter *g*. This stage consisted of four steps.

1. The visual stimuli (the Necker cube with randomly chosen contrast parameter *g*_*j*_) were displayed on the screen during time interval τ_*i*_, randomly chosen between 0.8 and 1.3 s.
2. After observing the stimulus on the screen, the subject analyzed its orientation and waited for the question of the experimenter.
3. Between subsequent demonstrations of the stimuli (Necker cubes), abstract pictures (AP) were exhibited during time η_*i*_, randomly chosen in the 5–7 s interval to divert attention and make the perception of the next image independent of the previous one.
4. *S* = 3 s after each Necker cube presentation, the experimenter asked the subject about her/his first visual impression about the cube orientation and according to his/her reply (“left” or “right”) recorded the result.
5. Steps (1–4) were repeated *N* = 200 times.

As in the first experimental set, the duration of each data acquisition in second set was also about 40 min. However, only *N* = 200 Necker cube images were presented, because in order for the subject to communicate its perceptions, abstract pictures showing time (η) had to be increased.

We want to emphasize that in the second set of experiments, the motor reaction of subjects was avoided since they did not need to press any key.

### 2.3. Preprocessing of the experimental EEG data

Before teaching the ANN to obtain a classification function, the preprocessing of the experimental EEG data was carried out. First, we reduced large amplitude artifacts found in the frontal cortex area, caused by eye blinks and movements. The form of the oculomotor EEG artifacts is known to be dependent on the type of eye movement in the horizontal/vertical direction in the presence/absence of angular momentum, in accordance the oculomotor artifacts can be classified into several types (Gratton et al., 1983; Jung et al., 2000). Eye movements are accompanied by changes in the electrical potential because the eyeball has an electric dipole moment formed by the potential difference between the retina and cornea of the eye (Jung et al., 2000; Ille et al., 2001, 2002; Hoffmann and Falkenstein, 2008). EEG signals recorded from subjects with open eyes can be represented as a linear combination of signals of the brain electrical activity and the interference caused by eye movements (Ille et al., 2001, 2002). It is a common practice to remove the interference (extraocular artifacts) by using a mathematical transformation of EEG and EOG signals with the method of Gram-Schmidt orthogonalization (Cheney and Kincaid, 2009). Particularly, in the experiments at hand the data from each electrode was processed using EOG obtained by means of the Gram-Schmidt orthogonalization procedure (Koronovskii et al., 2015).

Let *x*_*i*_(*t*) be the EEG signal of the *i*-th channel, *c*_ν_(*t*) and *c*_*h*_(*t*) are the EOG signals containing information about vertical and horizontal eye movements. These signals can be represented by the Gram-Schmidt orthogonalization procedure:

(1) $$\begin{array}{r} x_{i}^{\prime }\left(t\right)=x_{i}\left(t\right)-c_{\nu }^{0}\left(t\right)\overset{t_{1}+T}{\underset{t_{1}}{\int }}c_{\nu }^{0}\left(t^{\prime }\right)x_{i}\left(t^{\prime }\right)dt^{\prime }, \end{array}$$

(2) $$\begin{array}{r} {\tilde{x}}_{i}\left(t\right)=x_{i}^{\prime }\left(t\right)-c_{h}^{0}\left(t\right)\overset{t_{1}+T}{\underset{t_{1}}{\int }}c_{h}^{0}\left(t^{\prime }\right)x_{i}^{\prime }\left(t^{\prime }\right)dt^{\prime }, \end{array}$$

where ${\tilde{x}}_{i}\left(t\right)$ is the signal after oculomotor artifacts filtration and *t* ∈ [*t*_1_, *t*_1_ + *T*], where *t*_1_ is the starting time and *T* is the interval duration. Signals $c_{\nu }^{0}\left(t\right)$ and $c_{h}^{0}\left(t\right)$ are the normalized “reference” EOG signals corresponding to the vertical and horizontal eye movements, respectively (Ille et al., 2001; Joyce et al., 2004):

(3) $$\begin{array}{r} c_{\nu }^{0}\left(t\right)=\frac{c_{\nu }\left(t\right)}{\left||c_{\nu }\left(t\right)|\right|},\;c_{h}^{0}\left(t\right)=\frac{c_{h}\left(t\right)}{\left||c_{h}\left(t\right)|\right|}, \end{array}$$

where

(4) $$\begin{array}{r} \;||c_{v,h}\left(t\right)||=\sqrt{\overset{t_{1}+T}{\underset{t_{1}}{\int }}{\left(c_{v,h}\left(t\right)\right)}^{2}dt}. \end{array}$$

**Figure 3:**
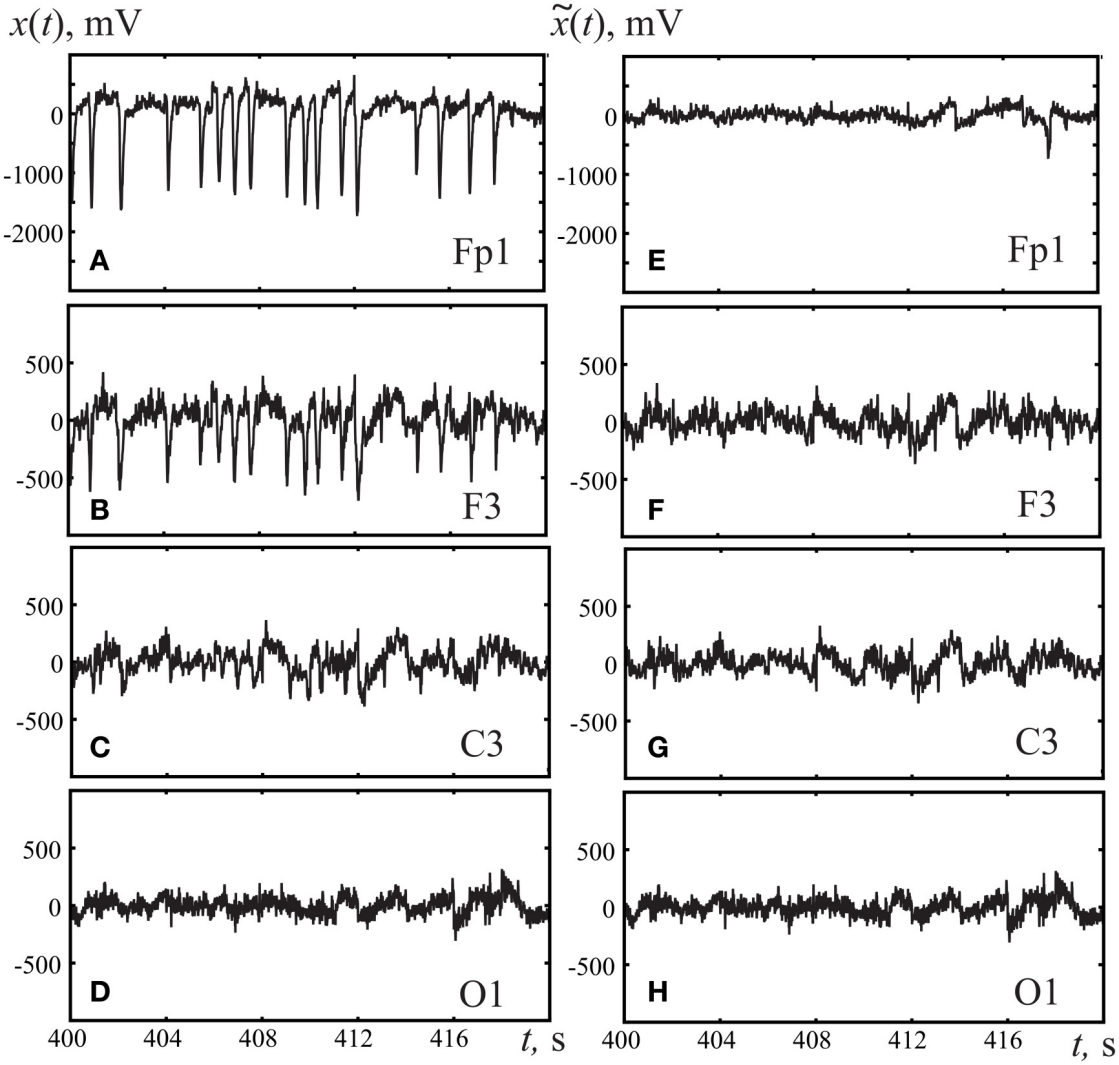
Typical EEG fragments registered in frontal cortex **(A,E)** Fp1, **(B,F)** F3, **(C,G)** motor cortex C3, and **(D,H)** occipital cortex O1. **(A–D)** corresponds to original registered EEG signals, **(E–H)** to the EEG signal after removing artifacts using the Gram-Schmidt procedure.

The orthogonalization procedure Equations (1–4) was performed on the signals from all 19 registered EEG channels. Figure 3 illustrates the application of the Gram-Schmidt orthogonalization procedure to remove the oculomotor artifacts caused by the horizontal and vertical movements of the eyeballs as well as by blinking. Figures 3A–D presents the initial EEG signals for different channels, clearly showing that the eye movement artifacts were more pronounced in the frontal channels than the occipital ones. Figures 3E–H exhibits EEG signals for the same channels after the Gram-Schmidt orthogonalization procedure. The comparison of Figures 3A,B,E,F underlines that the Gram-Schmidt orthogonalization method is an effective tool for removing oculomotor artifacts. The overall behavior of the EEG signal after applying the Gram-Schmidt procedure to remove oculomotor artifacts does not exhibit significant changes from the original form (cp. Figures 3D,H).

Single *T*-second duration trials corresponding to 250*T* samples, according to the 250-Hz sampling frequency of the data acquisition system “Encephalan-EEGR-19/26,” were extracted from all EEG data sets of each subject. These single trials *s*_*p*_(*t*) (*p* = 1, …*P*), where *P* is the number of channels of the EEG recording, were chosen to start at each stimulus onset, i.e., at the beginning of the presentation of each Necker cube with the contrast parameter *g*, and ended *T* seconds after the stimulus onset. These time intervals of *T* duration are marked by the red brackets in Figure 2. So, we extracted the EEG signal for further analysis during time intervals which directly correspond to the processes of visual perception and decision-marking on the left or right cube orientation. After that, all extracted trials for every subject were sorted according to his/her impression about the cube orientation (left or right key). Finally, the recorded time series from each EEG electrode were scaled to the interval [−1, 1].

**Figure 4:**
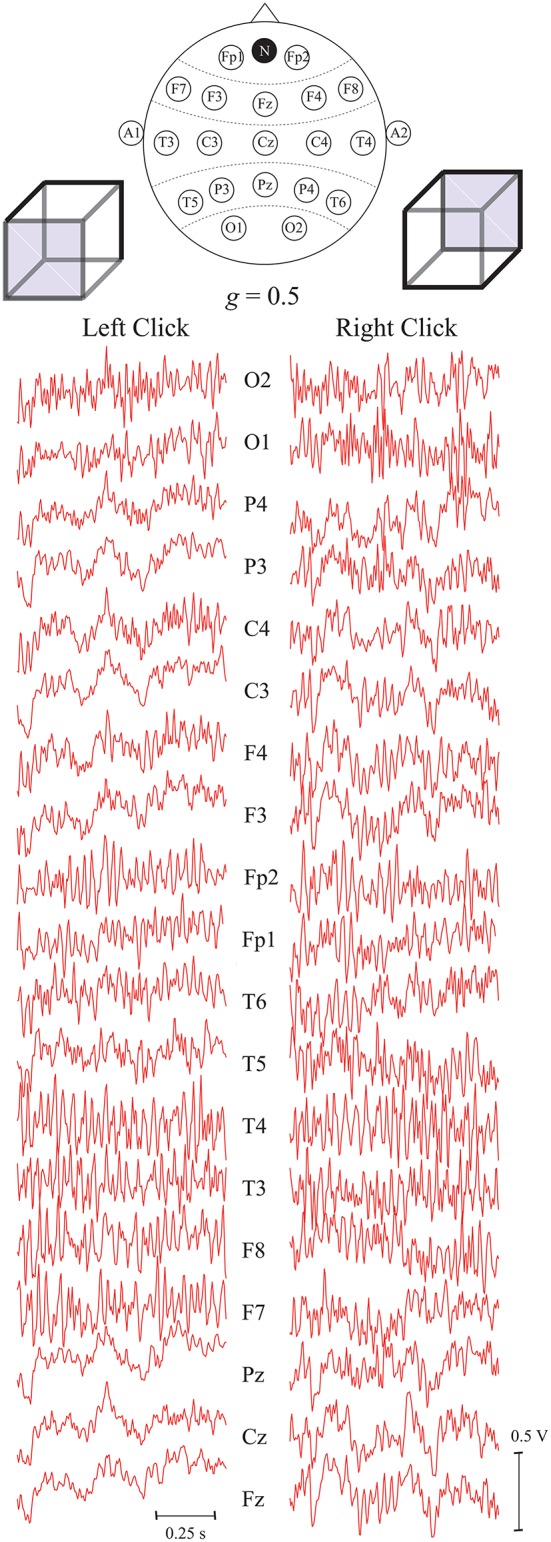
Typical EEG trials of one subject after preprocessing, related to two different interpretations of the Necker cube orientation with contrast parameter *g* = 0.5, recorded from different electrodes during *T* = 1 s after stimulus presentation. The top panel represents the international 10–20 scheme of *P* = 19 electrodes. The electrodes A_1_ and A_2_ are the reference ones and N is the ground electrode.

Figure 4 demonstrates typical EEG trials of one of the subjects from all 19 registered electrodes after the preprocessing procedure in the first set of experiments, i.e., with key pressing. The electrode positions are shown in the international 10–20 scheme on the top of the figure. These EEG traces of *T* = 1 s duration were recorded immediately after presentation of the Necker cube with the contrast parameter *g* = 0.5. The traces in the left and right panels correspond respectively to the left- and right-oriented cubes. The trials of 12 subjects were used as initial database to extract specific features from the time series using the ANN time domain technique.

### 2.4. Architecture of artificial neural network and description of classification algorithm

An artificial neural network (ANN) consists of a number of artificial neurons interconnected with each other by synaptic weights to form a net. Many possible ANN architectures can be used for pattern recognition. For example, a spiking neural network (SNN) simulates realistic neuronal behavior because it takes into account main properties of neurons, such as spike-timing-dependent plasticity. This type of ANN has proved to be an effective tool for pattern recognition (Masquelier et al., 2009; Grassia et al., 2017), but it is usually hardware-based and requires specific software/hardware platforms. In this study, we employ a class of ANN known as a *multilayer perceptron* (MLP), since it does not require a specific hardware and shows high capabilities while performing different computational tasks including pattern recognition. Also, MLP is much easier for practical implementation and therefore is widely used for many applications including the classification problem (Duda and Hart, 1973; Haselsteiner and Pfutscheller, 2000; Fontoura da and Cesar, 2001; Garrett et al., 2003; Dias et al., 2007; Hasan et al., 2015).

An important characteristic feature of the MLP is that a signal propagates in a forward direction only (feedforward network) from left to right on a layer-by-layer basis (Haykin, 2008). In our case, the classification problem consists in the recognition of two different brain states corresponding to the perception of the bistable Necker cube as left-oriented or right-oriented.

**Figure 5:**
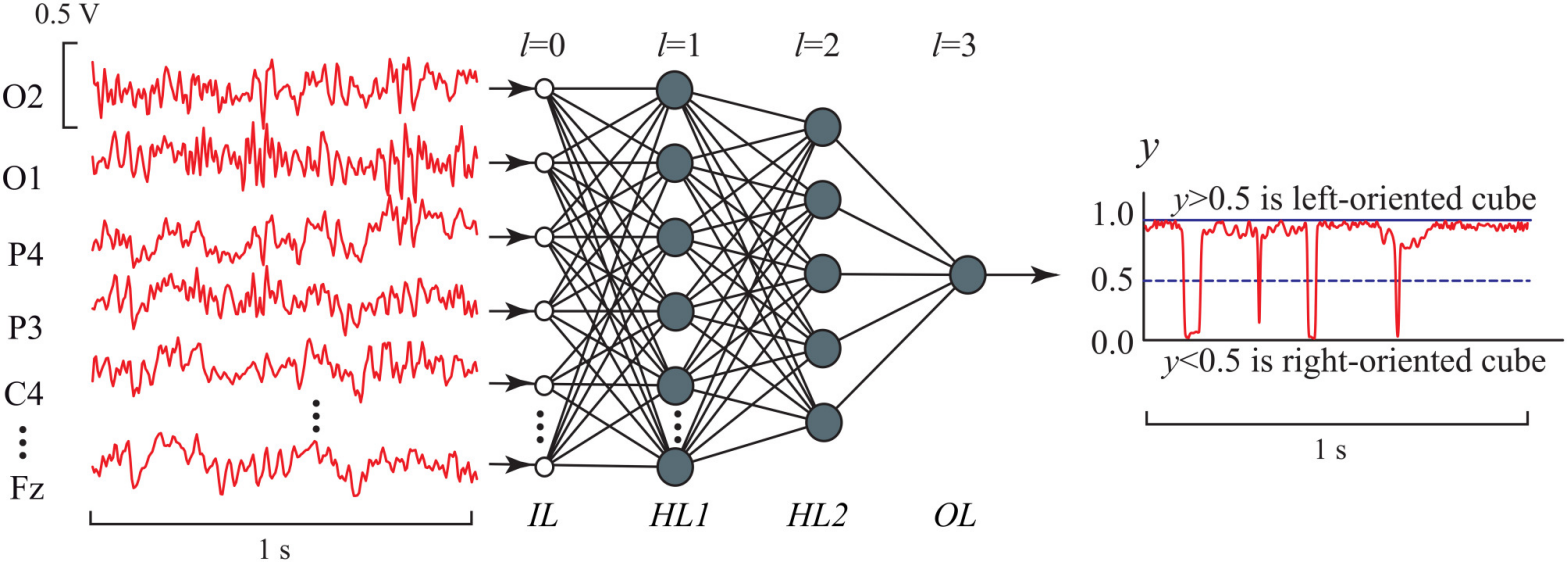
MLP architecture with two hidden layers in EEG signal classification. *IL* (*l* = 0) is the input layer, *HL*1 and *HL*2 are the first (*l* = 1) and second *l* = 2) hidden layers, respectively, which nodes (artificial neurons) are characterized by nonlinear activation function given by Equation (6), and *OL* is the output layer (*l* = 3) consisting of one artificial neuron with the same activation function. The number of inputs is *H*^0^ = *P* = 19, the numbers of nodes in the hidden layers are *H*^1^ = *P* and *H*^2^ = 5, respectively, and the number of output nodes is *H*^3^ = 1.

Figure 5 shows the ANN architecture of MLP used in our analysis for EEG signal classification. The ANN had input layer *IL*, two hidden layers, *HL1* and *HL2*, and output layer *OL*. The input layer *IL* contained *P* = 19 inputs, one for each of 19 EEG channels. For every *p*-th (*p* = 1, 2, …, 19) input we used the functional EEG signal *s*_*p*_(*t*) with 1-s duration (250 samples) from *p*-th channel registered for the case of left- or right-oriented cube interpretation (the examples of input data are shown Figure 4). The signal from each input was fed to all computational nodes in the first hidden layer *HL1* with *H*_1_ artificial neurons. The resulting output signal from *HL1* entered to the second hidden layer *HL2* with *H*_2_ neurons of the same type. Finally, the output signal from *HL2* entered to a single neuron in the output layer *OL*. Since our classification problem was the recognition of two brain states using the 19-channel EEG data set, the ANN contained only one output neuron, which output value should have classified the current brain state to either left- or right-oriented cube interpretation.

The ANN evolution is described by the following mathematical model (Yao, 1999)

(5) $$\begin{array}{r} u_{i}^{l}\left(t\right)=F^{l}\left(\overset{H^{l-1}}{\underset{p=1}{\sum }}w_{pi}^{l}u_{p}^{l-1}\left(t\right)-\theta_{i}^{l}\right), \end{array}$$

where *H*^*l*^ is the number of neurons in the *l*-th layer (a layer with *l* = 0 is supposed to be the input layer), $u_{i}^{l}\left(t\right)$ is the output signal of the *i*-th neuron belonging to the *l*-th layer [$u_{i}^{0}\left(t\right)$ being the signals from analyzed EEG channels], ${\text{W}}^{l}=\left\{w_{pi}^{l}\right\}$ is the weight matrix of the *l*-th layer of dimension (*H*^*l* − 1^ × *H*^*l*^), and $w_{pi}^{l}$ (*p* = 1, …, *H*^*l* − 1^, *i* = 1, …, *H*^*l*^) are the synaptic weights of input signals for the *i*-th neuron in the *l*-th layer, $\Theta^{l}=\left\{\theta_{i}^{l}\right\}$ is the threshold vector for neurons in the *l*-th layer, and

(6) $$\begin{array}{r} F^{l}\left(\eta \right)=f\left(\eta \right)=\frac{1}{1+\exp\left(-\eta \right)} \end{array}$$

is the nonlinear logistic activation function for neurons in the hidden and output layers *l* = 1, 2, 3.

A class of recognized objects can be characterized by the mean squared value of output signal $u\left(t\right)=u_{1}^{3}\left(t\right)$, as follows

(7) $$\begin{array}{r} y=\sqrt{\frac{1}{T}\overset{T}{\underset{0}{\int }}{\left(u\left(t\right)\right)}^{2}dt}. \end{array}$$

Since the input signals $u_{p}^{0}\left(t\right)$ are trials *s*_*p*_(*t*_*i*_) (*p* = 1, …*P*, *t*_*i*_ = *iΔt*, *i* = 1, …*N*) with the length *T* consisting of *N* = 250 samples (*T* = 1 s, Δ*t* = *T*/*N*), Equation (7) can be rewritten in the form

(8) $$\begin{array}{r} y=\sqrt{\frac{1}{N}\overset{N}{\underset{i=1}{\sum }}{\left(u\left(t_{i}\right)\right)}^{2}}. \end{array}$$

For the left-oriented Necker cube perception, the mean squared value of the output signal is supposed to be *y* ≥ 0.5 and for the right-oriented cube *y* < 0.5.

The unknown matrices **W**^*l*^ and vectors Θ^*l*^ can be obtained during the learning process by minimizing the classification error criterion:

(9) $$\begin{array}{r} \mu =\sqrt{\frac{1}{K}\overset{K}{\underset{k=1}{\sum }}{\left(d_{k}-y_{k}\right)}^{2}}, \end{array}$$

where *K* is the total number of objects in the training set, *y*_*k*_ is the mean squared value of the output signal calculated for the *k*-th object using Equation (8), *d*_*k*_ is a desired output value of *y*_*k*_ which we wish the MLP to learn (*d*_*k*_ = 1 corresponds to the left-oriented cube perception and *d*_*k*_ = 0 to the right-oriented one). To find unknown parameters of ANN, we used the Levenberg-Marquardt algorithm (LMA) (Strutz, 2016). By differentiating the error criterion Equation (9) with respect to the unknown parameters, the LMA method yields better results in comparison with other optimization methods, but requires more computational time to determine the unknown parameters. For the learning process, we created a data set consisting of 70 single trials with 1-s duration (250 samples) randomly selected from EEG records obtained from one volunteer (see section 2.3). This data set consisted of 35 trials for each orientation of the Necker cube images with different contract parameters *g*. For more reliable assessment of the result of ANN learning, we repeated the training procedure for a total of a 1,000 learning cycles. As a consequence, we obtained 1,000 ANNs with different parameters and different error classification values μ.

To estimate ambiguous images classification precision, we calculated *recognition accuracy* ρ defined as

(10) $$\begin{array}{r} \rho =\frac{N_{p}}{N}\times 100\%, \end{array}$$

where *N*_*p*_ is the number of true classified cubes and *N* is the total number of analyzed Necker cube images.

Finally, for further analysis we chose the ANN with the smallest classification error μ characterized by the highest accuracy ρ, to be the best ANN for classification. The procedure of ANN learning was implemented for each volunteer in order to find his/her optimal ANN topology with the highest recognition accuracy.

### 2.5. Wavelet analysis

The set of EEG signals was analyzed with the help of the continuous wavelet transformation. For each *m*-th observation of the Necker cube, the wavelet energy spectrum $E_{n}^{m}\left(f,t\right)=W_{n}^{m}{\left(f,t\right)}^{2}$ was calculated for each EEG channel *X*_*n*_(*t*) in the frequency range *f* ∈ [1, 35] Hz and approximately 3-s time interval, including 1-sec intervals before and after the presentation. Here, $W_{n}^{m}\left(f,t\right)$ is the complex-valued wavelet coefficients calculated as (Hramov et al., 2015)

(11) $$\begin{array}{r} W_{n}^{m}\left(f,t\right)=\sqrt{f}\overset{t+4/f}{\underset{t-4/f}{\int }}X_{n}\left(t\right)\psi^{*}\left(f,t\right)dt, \end{array}$$

where *n* = 1, …, *N* is the EEG chanel number and “^*^” defines the complex conjugation. The mother wavelet function ψ(*f, t*) is the Morlet wavelet, often used for the analysis of neurophysiological data, defined as (Hramov et al., 2015)

(12) $$\begin{array}{r} \psi \left(f,t\right)=f^{1/2}\pi^{1/4}{\text{e}}^{j\omega_{0}f\left(t-t_{0}\right)}{\text{e}}^{f{\left(t-t_{0}\right)}^{2}/2}, \end{array}$$

where ω_0_ = 2π is the center frequency of the Morlet wavelet. The obtained surfaces $E_{n}^{m}\left(f,t\right)$ were calculated for *M* = 300 presentations (150 left-oriented and 150 right-oriented). The values of $E_{n}^{m}\left(f,t\right)$ were then averaged over occipital EEG signals and over the number of presentations associated with left-oriented and right-oriented cubes, separately. As the result, for each subject the values 〈*A*_*L*_(*f, t*)〉 and 〈*A*_*R*_(*f, t*)〉 (subindices *L* and *R* refer to left- and right-oriented cubes, respectively) reflected the averaged time-frequency EEG structure associated with left- and right-oriented cube interpretations. In order to qualitatively characterize the difference between the averaged wavelet spectra, we took into consideration coefficient 〈Δ*A*〉 defined as follows

(13) $$\begin{array}{r} \langle \Delta A\rangle =\overset{f_{2}}{\underset{f_{1}}{\int }}\overset{t_{2}}{\underset{t_{1}}{\int }}\left(\langle A_{L}\left(f,t\right)\rangle -\langle A_{R}\left(f,t\right)\rangle \right)df\;dt. \end{array}$$

Here, the integration was performed over the frequency band 1–35 Hz and the 3-s time interval including 1-s intervals before and after the presentation.

## 3. Results

### 3.1. Optimal method parameters and ANN topology

The most important point in our research was the choice of an optimal ANN architecture to solve the classification problem. Indeed, if the ANN architecture is too simple, i.e., the number of neurons in hidden layers are inadequately small, the problem cannot be resolved. On the contrary, an excessively complex ANN structure requires a very long learning time. Therefore, the ANN topology (number of neurons in hidden layers) should be optimized in order to obtain a reasonable accuracy in the EEG classification problem during perception of ambiguous images.

**Figure 6:**
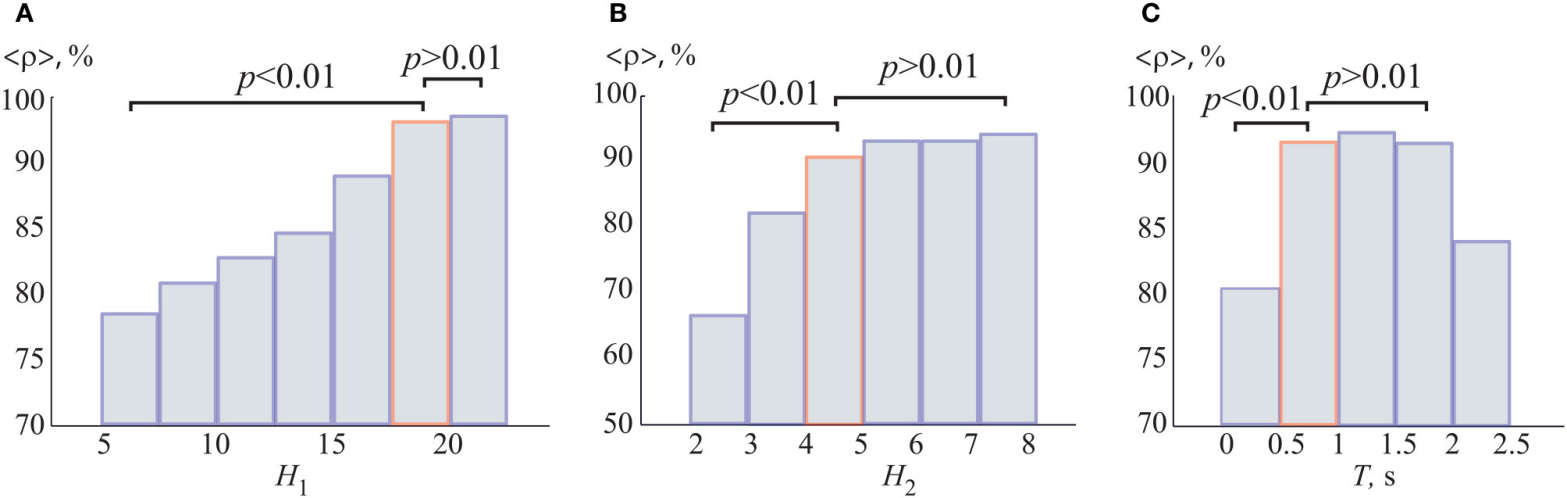
Recognition accuracy as a function of the number of neurons **(A)** *H*_1_ in the first hidden layer (for *H*_2_ = 5) and **(B)** *H*_2_ in the second hidden layer (for *H*_1_ = 19). We used single EEG trials with *T* = 1 s duration (250 samples). **(C)** Recognition accuracy as a function of duration *T* of single EEG trials. The accuracies were averaged over all 1,000 training ANNs. All data was averaged all participants in the group. *p*-values were calculated via paired *t*-test. The boxes highlighted in orange correspond to the optimal parameters.

In order to prove that the chosen ANN architecture was optimal from the classification problem, we calculated the dependencies of the recognition accuracy ρ on the number of neurons in the hidden layers HL1 and HL2, shown in Figures 6A,B. The accuracy ρ was averaged over all 1,000 training ANNs and over all participants. One can see that the average accuracy grew rapidly as the number of neurons in the hidden layers was increased. Specifically, the paired *t*-test statistical analysis showed that the accuracy significantly increased when the number of neurons in layers HL1 and HL2 went from 5 to 17 and from 2 to 5, respectively. A further increment in the number of neurons did not lead to an additional augmentation of the accuracy. According to this result, the optimal parameters of the neural network were set to *H*_1_ = *P* = 19 and *H*_2_ = 5, respectively. One can see that for the chosen parameters of the optimal ANN topology, the averaged accuracy ρ exceeded 90%.

Another important issue was how to optimize the duration *T* of EEG trials for maximum recognition accuracy. In the previous work (Merk and Schnakenberg, 2002), the experimentally measured typical duration of one of the percepts of the Necker cube was found to be approximately 1 s. Here, we analyzed the recognition accuracy ρ vs. the duration *T* of single EEG trials used for classification. Figure 6C shows the dependence of the recognition accuracy ρ on the duration *T* in the range of [0.1, 2.25] s which corresponds to [25, 562] samples. The accuracy ρ was averaged over all 1,000 training ANNs. One can see that the accuracy reached its maximum in the range of [0.75, 1.75] s. Therefore, we chose the duration of the EEG trials for further analysis to be equal to *T* = 1 s. Also, it should be noted that, when working in on-line regime (for example, for brain-computer interfaces development; Bell et al., 2008; Maksimenko et al., 2017; McFarland et al., 2017), the analysis of short time series will be more preferable.

### 3.2. Recognition and classification of multistable brain states using EEG data

The development of our classification algorithm was started with the training of ANNs for each subject who participated in our experiments. The training data set was formed individually for every participant and the optimal set of ANN parameters $\Gamma_{r}=\left({\text{W}}_{r}^{1},{\text{W}}_{r}^{2},{\text{W}}_{r}^{3},\Theta_{r}^{1},\Theta_{r}^{2},\theta_{r}^{3}\right)$ was obtained for brain states classification of subject *r* = 1, …12.

**Figure 7:**
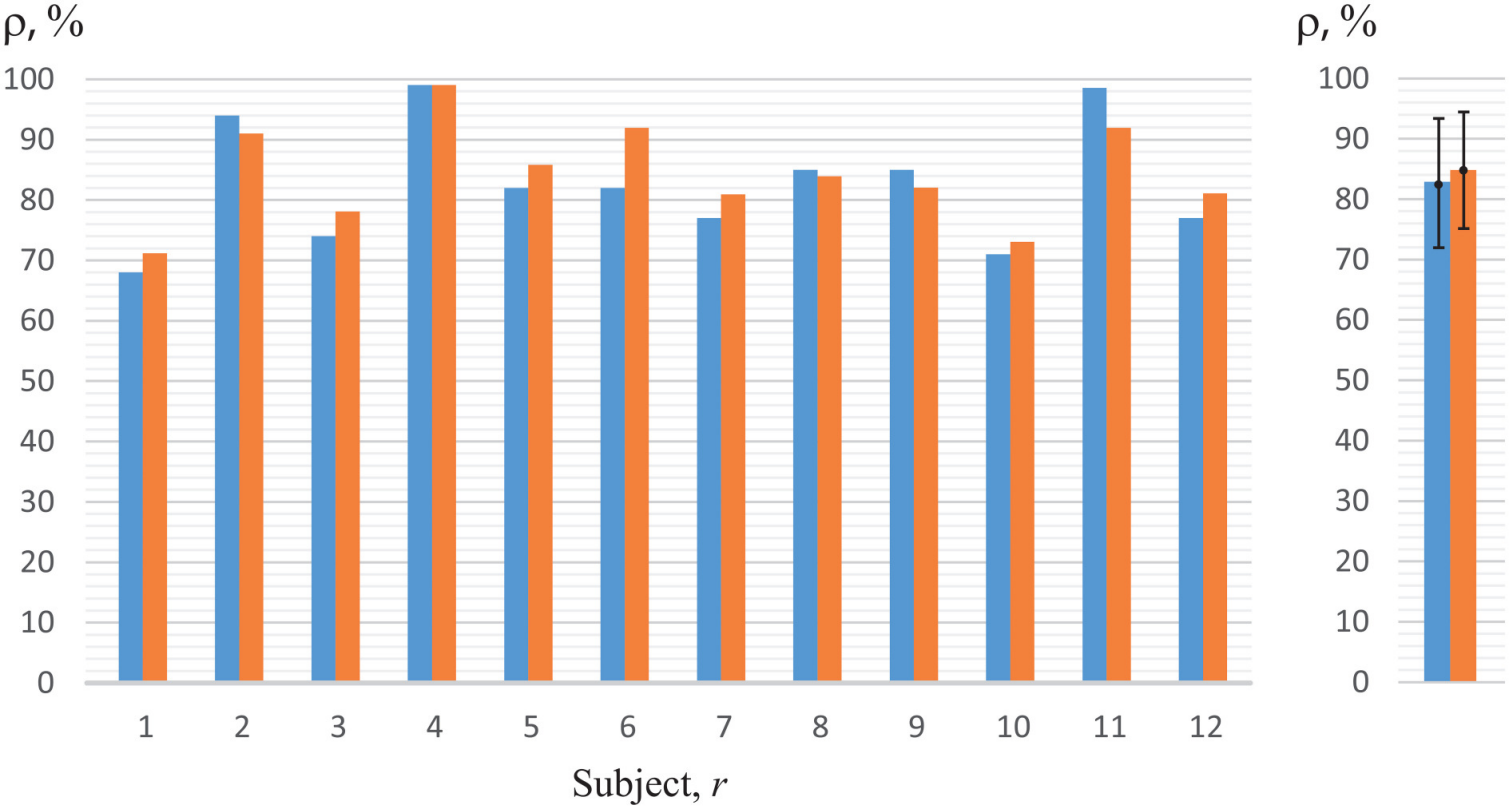
Recognition accuracy for all of the 12 subjects. The left-hand blue columns represent accuracy for each subject, using ANN trained on his/her own EEG (*h* = *r*) obtained in the first set of experiments (with key pressing). The right-hand orange columns show accuracy for the second set of experiments (without key pressing). The right panel represents the data averaged over all subjects under study.

Now, we will analyze the experimental data obtained in the first set of experiments (with key pressing) (Figure 1A). The recognition accuracy of the brain states classification during visual perception of ambiguous images (left-/right-oriented perception) for each of the 12 subjects, used for training ANN, are shown in Figure 7. To analyze the classification accuracy we took the part of the EEG which was not used for training, i.e., about 330 EEG trials of the registered brain states after image demonstration. In this case, the mean classification accuracy for all of the 12 subjects was close to 82.6 ± 10.7% (mean ± S.D.) (left blue column in the right panel of Figure 7). The recognition accuracy for every subject, shown in the blue left columns in the left panel of Figure 7, varied between 68 and 98% for different subjects.

Practically the same recognition accuracy was calculated by analyzing experimental data obtained in the second set of experiments (without key pressing). The recognition accuracy of the brain states classification for each of the 12 subjects is shown in Figure 7 when the experimenter asked the subject of how he/she interpreted the Necker cube, and then made the corresponding note in the presentation software according to the answer. As in the previous case, to analyze the recognition accuracy we took the part of the EEG which was not used for training, i.e., about 130 EEG trials of the registered brain states after image demonstration. The mean classification accuracy for all of the 12 subjects was close to 87% (right orange column in the right panel in Figure 7), while the recognition accuracy for every subject, shown in the right orange columns in the left panel of Figure 7, varied between 71 and 98% for different subjects.

Thus, the comparison of the results for classification of the brain states obtained in two different sets of experiments (with and without key pressing) demonstrated almost identical results for the same subject. The quality of recognition of the brain states in the group of 12 subjects was at the level of 82–84% in both sets of experiments. Therefore, we can conclude that the motor reaction had no effect on the classification quality. In the next stage of this study, we will discuss the results of the second set only, i.e., the experiments which did not include real or imaginary motor activity.

### 3.3. Cross-subject classification of brain states using EEG data of different subjects

**Figure 8:**
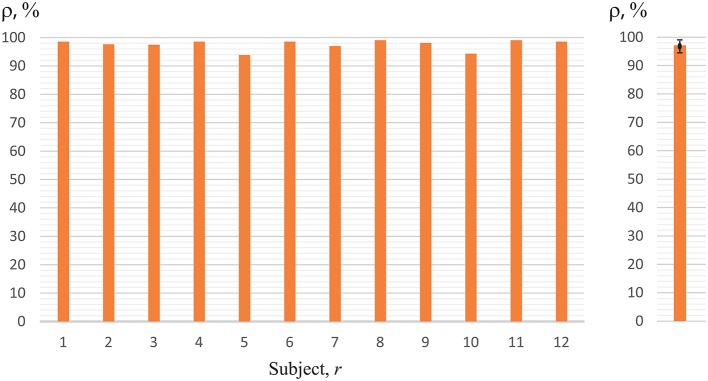
Recognition accuracy using ANN trained on subject 4 (*h* = 4) for all of the 12 subjects. The right panel represents the data averaged over all subjects under study.

It is remarkable that one of the subjects (*r* = 4) demonstrated very high recognition accuracy in classification of image perception, which reached 98%. When we applied the ANN trained on this subject to the analysis of the EEG data of other subjects, we obtained much higher accuracy than when we used the ANNs trained on their own data. These results are shown in Figure 8. Using ANN with parameters Γ_4_ evaluated for subject *r* = 4 the accuracy of classification was close to 95–98% for almost all subjects, except for subjects *r* = 5 and *r* = 10, who demonstrated ρ < 95%. Thus, we can conclude that the features of EEG patterns corresponding to the perception of left- or right-oriented cubes were typical for all subjects, and a single ANN trained on the EEG data set of one person can classify with high accuracy the corresponding brain states of a large group of people.

**Figure 9:**
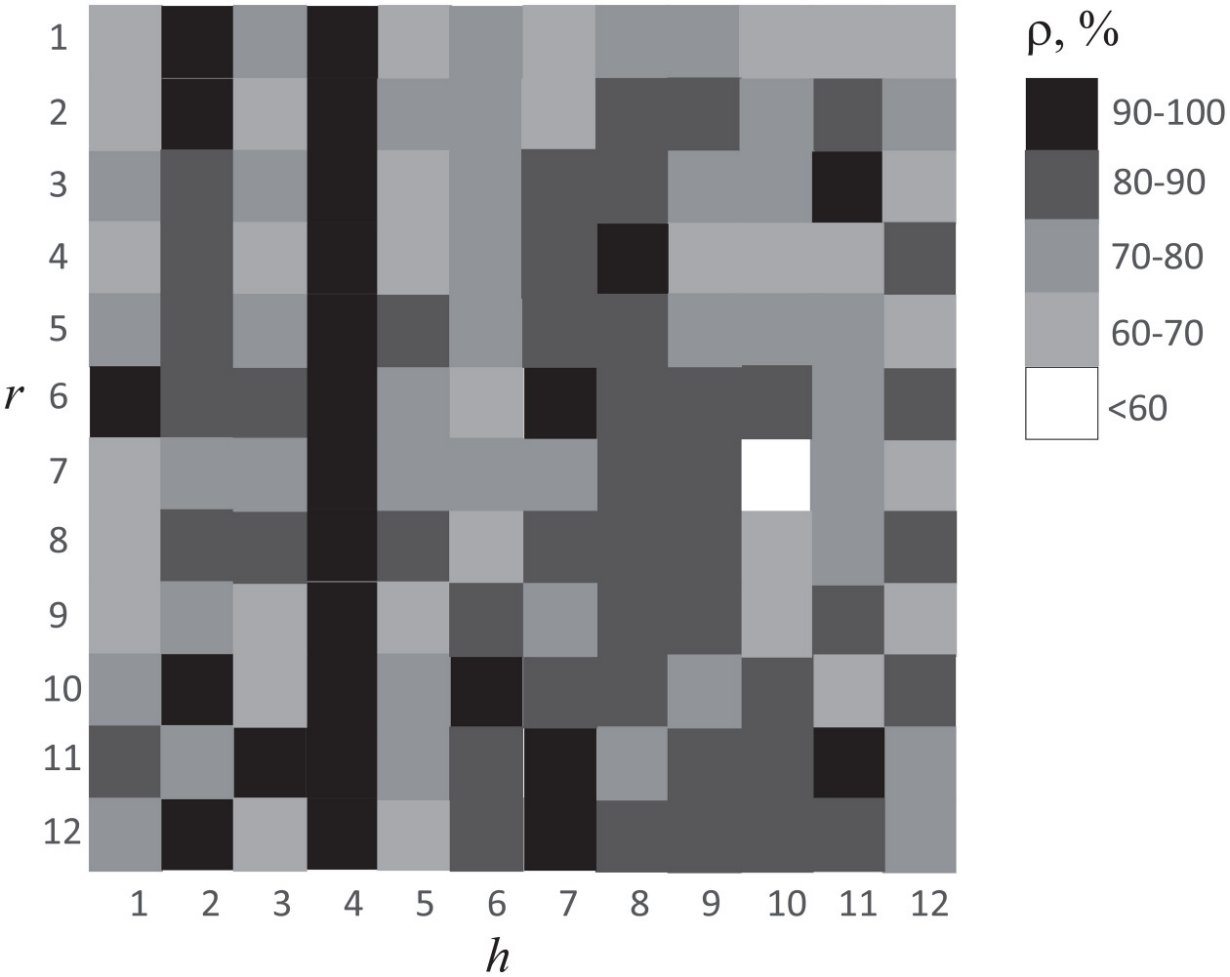
Accuracy of cross-subject classification of brain states of subject *r* using ANN trained on EEG data of subject *h*.

Reasonable accuracy was obtained when classification was made using ANN of any subject *h*. The results are shown in Figure 9, where we plot the accuracy of cross-subject classification of the EEG data of subject *r* = 1, …12 using ANN (with parameters Γ_*h*_) trained on subject *h* = 1, …12. One can see that recognition accuracy falls below 60% only in one case. Therefore, we can conclude that ANN trained on one subject gave good results on brain states classification of all other subjects. It should be noted that in Figure 9 data observed on diagonal *h* = *r* corresponds to the recognition accuracy presented in Figure 7. One can see that the subject *h* = 4 demonstrated the best results on classification of the whole group under study. The EEG patterns corresponding to the perception of left- or right-oriented cubes of this particular subject exhibited the most typical features making his EEG data set universal for identification and classification of the brain states.

**Figure 10:**
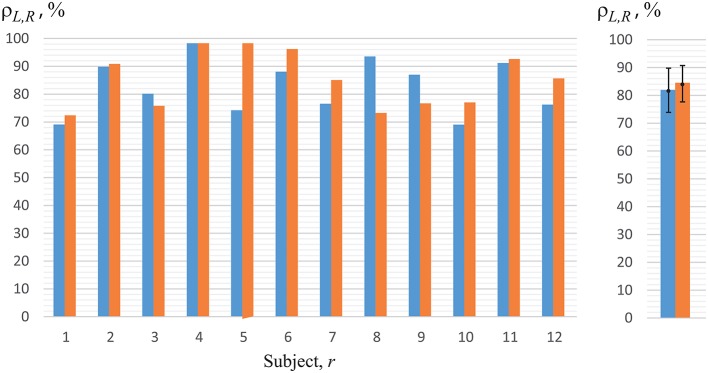
Recognition accuracy for left-oriented (left blue columns), ρ_*L*_, and right-oriented (right orange columns), ρ_*R*_, Necker cube perception for all of the 12 subjects using ANN trained on the EEG of the same subject (*h* = *r*). The right panel represents the data averaged over all persons under study.

Finally, in Figure 10 we present the results in perception recognition of left-oriented cubes (left blue columns) and right-oriented cubes (right orange columns) separately, using individual ANNs for each subject trained on his/her own EEG data (cp. with Figure 7). We should note that the mean accuracy in perception recognition of left-oriented (ρ_*L*_ = 82.0 ± 0.8) and right-oriented (ρ_*R*_ = 84.0 ± 0.6) cubes was insignificant. Besides, we observed that for several persons (*r* = 5, 8, 9, 12) the recognition accuracies of the right-oriented and left-oriented cubes perception differed by more than 10%. This fact can be explained by individual physiological features of the subjects, for example, ocular dominance or eye preference.

### 3.4. Results of time-frequency analysis

**Figure 11:**
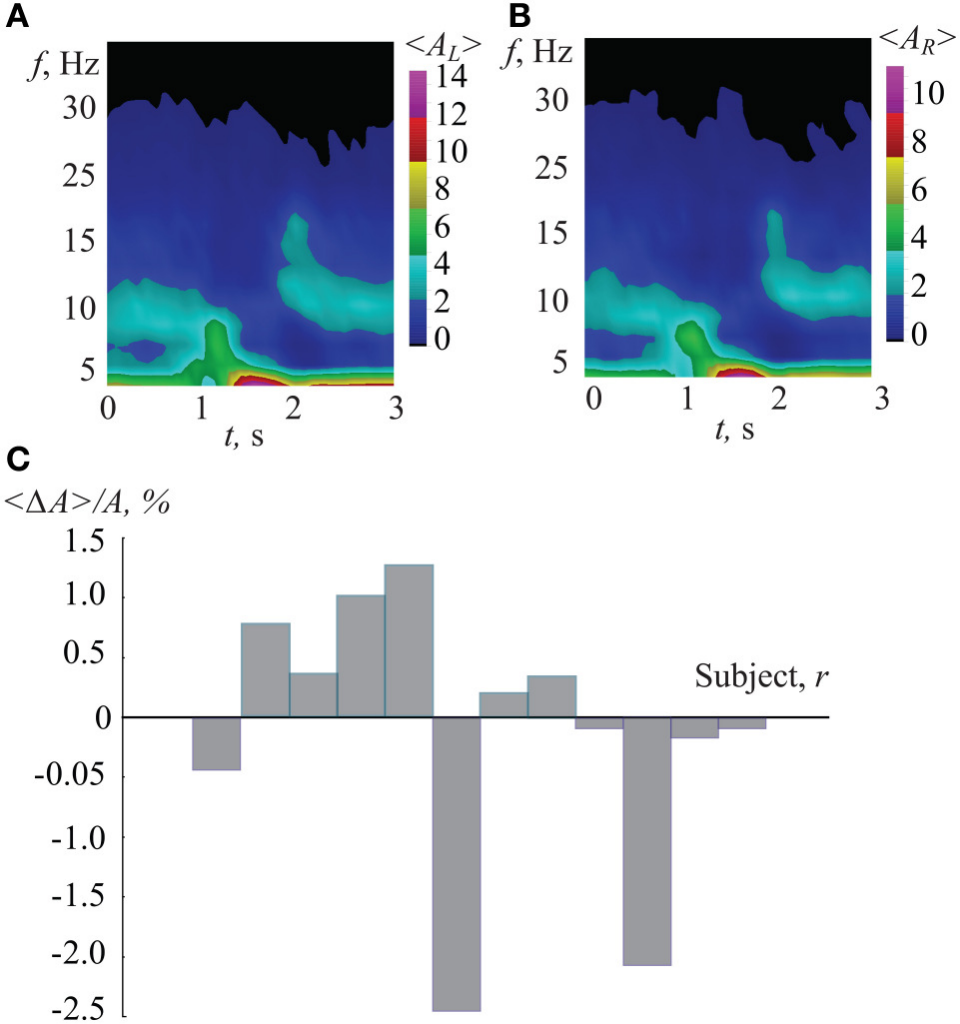
**(A,B)** Typical wavelet power spectra averaged over 150 EEG epochs corresponding to perception of Necker cubes (including 1-s intervals before and after cube exhibition), associated with **(A)** left- and **(B)** right-oriented cube interpretations. **(C)** Normalized comparison coefficient between wavelet spectra corresponding to different interpretations of the Necker cube, averaged over 150 epochs for each subject.

To compare the classification efficiency when using ANN with time-frequency analysis, we considered the wavelet spectra of EEG trials corresponding to different interpretations of the Necker cubes. The results of the wavelet transform analysis applied to our data are shown in Figure 11. In Figures 11A,B one can see the typical time-frequency structure of the EEG signals corresponding, respectively, to the left-oriented (〈*A*_*L*_(*f, t*)〉) and right-oriented (〈*A*_*R*_(*f, t*)〉) interpretations of the Necker cube. The presented spectra were obtained by averaging over 150 interpretations for each type. It is seen that both cases were characterized by the destruction of the α-rhythm associated with the brain's response to the visual stimuli (Klimesch, 2012; Ikkai et al., 2016). Also, the presented time-frequency plots do not show a significant difference in the time-frequency structure associated with left- and right-oriented interpretations.

In order to qualitatively characterize the difference between left- and right-oriented cube interpretations, coefficient 〈Δ*A*〉 was calculated for each of the 12 subjects. This coefficient normalized to the averaged spectrum is shown in Figure 11C. One can see that while some subjects demonstrated positive values of 〈Δ*A*〉, others had negative 〈Δ*A*〉. As a result, the difference in the time-frequency structure corresponding to different interpretations is insignificant. Therefore, the time-frequency analysis does not allow classification of the EEG patterns with respect to left- and right-oriented cube interpretations.

## 4. Discussion

In the previous sections we demonstrated close to 95% accuracy in classification of the EEG patterns during perception of ambiguous images by means of the classifier based on the artificial neuronal network. Now, we will discuss the important issue about possible classification of the measured motor preparation for a particular motor act (left/right key pressing).

Firstly, it should be noted that according to literature, EEG classification of the real and imaginary movements of the hand and, especially, fingers is a very complicated and non-trivial task for untrained subjects (Blankertz et al., 2007). For example, it has been demonstrated (Ferrante et al., 2015) that the existing motor act classification algorithms when applied to untrained participants do not always achieve good performances. However, one can expect that ANN being trained on the data of one subject will prove more successful at classification of the movement-related EEG trails of other subjects.

Secondly, in order to finally prove our statement, we performed additional cross-experiment analysis to exclude the effect of the motor preparation. In the first set of experiments and the preparation for the answer in the second set, we mathematically processed the experimental data as follows. (i) We used ANNs trained on the EEG data obtained in the first experiments (with key pressing) for classification of EEG trials recorded in the second experiments (without key pressing) for the same subject. (ii) Conversely, we applied ANNs trained on the EEG data obtained in the second experiment for classification of EEG trials recorded in the first experiment for the same subject. In both cases the recognition accuracies of cross-experimental classification was close to those obtained for each set of the experiments. The quality of recognition of the brain states in the group of all of the 12 subjects was at the level of ρ_12_ = 81.2 ± 11.2% for cross-classification case (i), and ρ_21_ = 87.1 ± 9.3% for cross-classification case (ii). Therefore, we can conclude that the motor act in the first set of experiments and the answer preparation in the second set of experiments had no effect on the classification results. The recognition accuracies ρ_12_ and ρ_21_ obtained with different experimental designs were close to each other. This means that the proposed ANN-based method gives correct results in classification of the perceptual brain states.

Another factor which could affect the validity of classification is the effect of eye movement and eye position. According to Einhäuser et al. (2004), the Necker cube is associated with differences in eye position and eye movements. We should note that it is mostly true in the case of prolonged observation. In our experimental design we tried to minimize these effects by choosing short time intervals for Necker cube demonstration (1.0–1.5 s). During this short time the subject could get only the first impression about the demonstrated object. Also, we drew the red dot at the center of the Necker cube image to focus the sight and prevent perception shifts due to the eyes movement. In this context, we assume that the effect of perception shifts due to eye-position was minimized in our studies.

From the analysis perspective of specific and universal features of the EEG brain states, our findings are very promising and can lead to new research. We have shown that the ANN-based method can be successfully applied for the study of the brain states associated with different interpretations of ambiguous images. We have demonstrated not only the ability of ANN to recognize EEG patterns corresponding to different Necker cube interpretations, but also the relevance of ANN for the detection of universal features of brain activity associated with different interpretations of bistable stimuli. The latter has been achieved by the application of ANN for the cross-subject classification. Namely, the ANN being trained on EEG trials data set of one subject has demonstrated high performance in the classification of analogous states in other subjects. This is surprising because the ANN is usually considered as a “black box” which is trained to effectively classify or detect features in those datasets for which it is initially trained. In this case, ANN is trained to learn very special features in order to provide maximal efficiency for the concrete data. Nevertheless, in our study the property of ANN to learn features invisible for eye, has been used to reveal fundamental aspects of bistable perception. In particular, the results of cross-subject classification provided the evidence that all participants did exhibit common brain states when perceiving left- or right-oriented Necker cube interpretations.

In neuroscience, the task of revealing fundamental aspects of brain dynamics always attracts a lot of attention. The features detection of brain activity is usually based on the analysis of the EEG signals time-frequency structure with the help of Fourier transform (Gotman et al., 1973) and adaptive wavelet (Hramov et al., 2015) transform. Along with these methods, there are also other approaches used for quantitative classification and feature extraction of the EEG patterns, such as e.g., discriminant analysis and independent component analysis (Makeig et al., 1996; Ungureanu et al., 2004; Hobson and Hillebrand, 2006). However, the wavelet-based methods give better results for classification and allocation of EEG patterns (Sitnikova et al., 2009, 2014; Nazimov et al., 2013) than other methods.

Previous studies of ambiguous figures using EEG trials, event-related potentials (ERP) and fMRI showed that perception of bistable images was accompanied by activation of specific brain areas and deactivation of others (Inui et al., 2000; Müller et al., 2005; Kornmeier et al., 2007). In particular, Kornmeier and Bach (2006) found a chain of ERP components during observation of a Necker lattice led to spontaneous perceptual reversals. They detected significant changes in the ERP in the occipital cortex (Oz electrode position).

Based on these related works, we considered the time-frequency structure of occipital EEG when the subjects exhibited left- and right-oriented interpretations of the Necker cube in order to understand the features of the related brain states. However, our results of wavelet decomposition of the EEG have not been able to reveal differences in the states associated with distinct Necker cube interpretations (see Figure 11).

**Figure 12:**
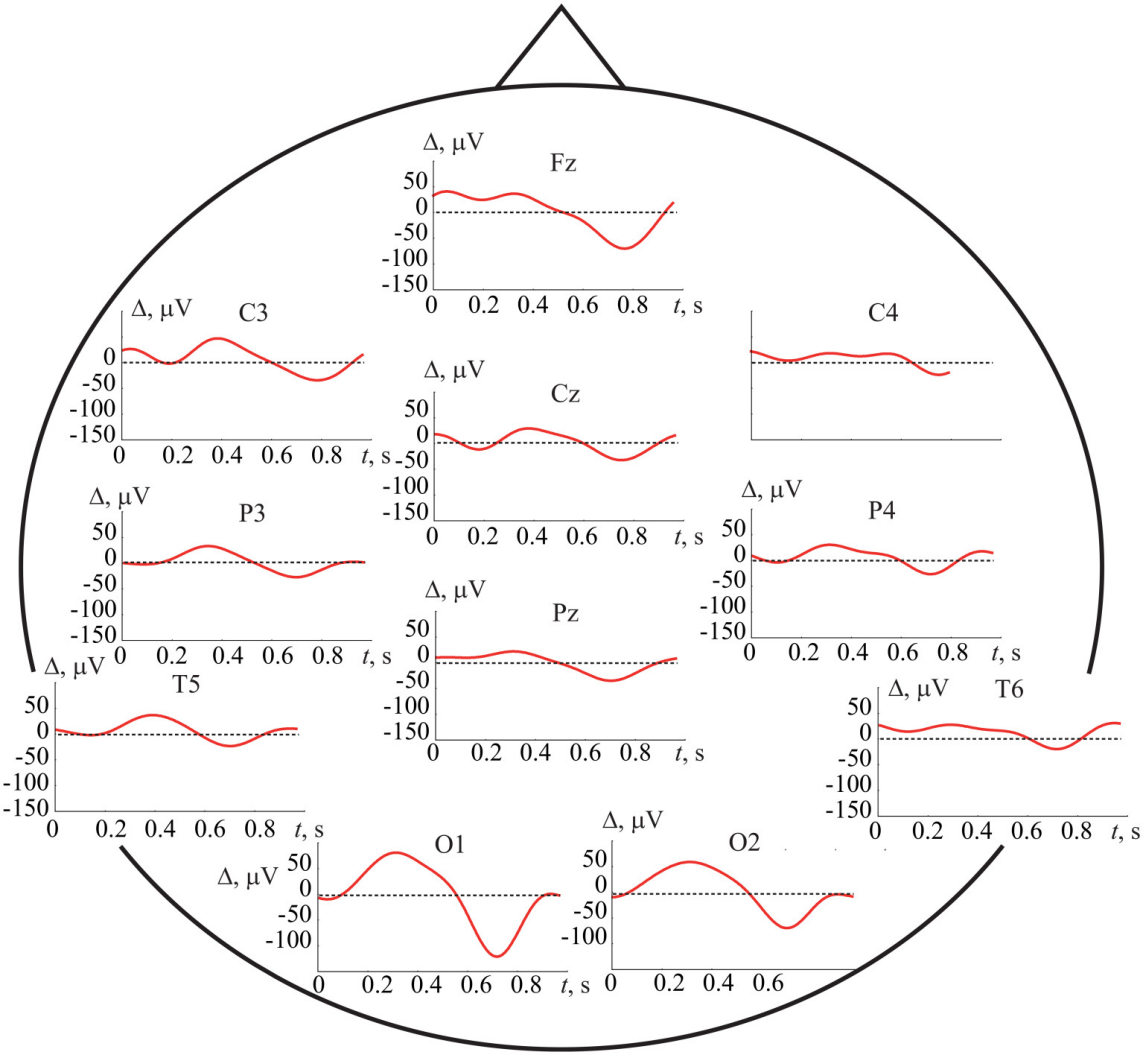
Averaged EEG difference traces Δ(*t*) for subject *h* = 4 recorded from 11 electrodes.

It was supposed that the waveform of the EEG signal contains the most pronounced features of left- and right-oriented interpretations of the Necker cube. In order to extract characteristic shapes of the EEG signals, we estimated the difference between ERPs corresponding to EEG trials associated with left and right cube orientations, recorded from different electrodes. For this purpose, we calculated the averaged EEG traces of *T* = 1 s duration corresponding to the perception of each type of the Necker cube for a particular subject, as follows

(14) $$\begin{array}{r} {\overset{-}{x}}_{p}^{L,R}\left(t\right)=\overset{N_{L,R}}{\underset{i=1}{\sum }}\left(s_{pi}\left(t\right)\right)/M_{L,R}, \end{array}$$

where *N*_*L,R*_ ≈ 100 is the number of perceptions of the left-(L)/right-(R) oriented cube and *p* = 1, …*P* (*P* = 19) is the EEG channel number. We observed high differences between averaged signals ${\overset{-}{x}}_{p}^{L,R}\left(t\right)$ from different electrodes. To characterize the features of the signal corresponding to left-/right-oriented cube perception, we defined the averaged EEG difference traces as $\Delta_{p}\left(t\right)={\overset{-}{x}}_{p}^{L}\left(t\right)-{\overset{-}{x}}_{p}^{R}\left(t\right)$. The averaged EEG difference traces from 11 electrode positions are presented in Figure 12 for subject *h* = 4 who exhibited the best results in the accuracy of cross-subject classification, i.e., who had the most pronounced EEG features identified by the ANN.

At first sight, remarkable variability Δ_*p*_(*t*) between EEG traces belonging to different Necker cube perceptions exists and can be revealed via the ERP analysis. However, closer examination revealed that the earliest significant component is positive difference Δ>0 in the time interval (0.2, 0.4) sec and negative difference Δ < 0 in (0.6, 0.8) s, most prominent in the occipital left (O1) and right (O2) locations. The second effect of the negative difference was more pronounced in the central frontal electrode positions (Fz). Similar trends were observed in the parietal, somatosensory, and temporal positions of the electrodes (see Figure 12), but they were less pronounced.

Having summarized the results in the detection of the brain states associated with left- and right-oriented interpretation of bistable images, we can conclude that the analysis of ERP evidences the difference between these two states appeared in occipital lobe. This result is in agreement with previous observations of Kornmeier and Bach (2006) who reported the responsibility of occipital area for perception. Surprisingly, the detailed time-frequency analysis of occipital EEG, based on wavelet decomposition, does not give any further information about distinctive features of these states, but, on the contrary, enhances their similarity.

In contrast to the previous approaches, the ANN applied in a cross-subject mode provides a strong evidence that the brain difference between the brain states associated with left- and right-oriented Necker cube interpretations does exist and can be considered as a universal phenomenon for different subjects. Moreover, compared to ERP study, the ANN finds these differences very significant and therefore can be applied to classify individual trials.

It should be noted that along with differences in operation of ANN and ERP, there are some fundamental similarities in the results obtained by these methods. In particular, according to ERP we can conclude that the most significant EEG channels for classification belong to the occipital cortex. Indeed, our preliminary calculations with the same ANN topology for 4 subjects (*h* = 2, 3, 4, 12) showed that the accuracy of brain states classification using only EEG channels in the occipital region (*P* = 6: O1, O2, P3, P4, Cz, Pz) is not significantly worse than using the complete set of channels (*P* = 19). However, the choice of optimal EEG channels requires further investigation.

## 5. Conclusion

In this paper, we have proposed the use of an artificial neuronal network for classification and automatic recognition of human brain states associated with the perception of ambiguous images. From obtained experimental data, we optimized the ANN architecture and achieved up to 95% accuracy in the classification of the EEG patterns during perception of ambiguous images. We have found particular features of the EEG patterns corresponding to different interpretations of the Necker cube, typical for all subjects, so that a single ANN trained on the EEG data set of one person can classify with high quality the corresponding brain states of a large group of people. Two sets of experiments, with key pressing and without key pressing, have demonstrated that the motor activity (real or imaginary) had no influence on the results in the cube classification.

We firmly believe that the significance of our results is not limited to visual perception of the Necker cube images. We are sure that the proposed experimental approach and developed computational technique based on the ANN can be applied for studying and classifying different brain states using EEG and MEG data, and can be useful in future research in the field of cognitive and pathological brain activity. The developed approach provides a solid experimental basis for further understanding of brain functionality. The rather simple way to quantitatively characterize brain activity related to perception of ambiguous images seems to be a powerful tool, which may be used in neurotechnology, e.g., for the brain-computer interface (BCI) task (Bell et al., 2008; McFarland et al., 2017) and in medicine for diagnostic and prognostic purposes (Ovchinnikov et al., 2010; Maksimenko et al., 2017). The efficiency of BCI is known to be defined by the ability of the operator to generate certain stable EEG patterns. This means that the BCI is affected by inter-subject variability (Ferrante et al., 2015). In this respect, our results suggest possibility for the development of an unified ANN-based classifier, which in turn can be used for building BCI for multiple and untrained persons (Blankertz et al., 2007). We expect that the results of this work will be interesting and useful for scientists carrying out interdisciplinary research at the cutting edge of physics, mathematics, neurophysiology, and medicine.

## Author contributions

AH and AP: Conceived the study and performed the analysis of experimental data; AR and VG: Provided the experimental studies; AH, AK, and AP: Prepared the manuscript; SP and VYM: Developed the artificial neuronal network model; MZ and AK: Provided the preprocessing of EEG; VAM: Calculated the wavelet spectra and provided the statistical analysis.

### Conflict of interest statement

The authors declare that the research was conducted in the absence of any commercial or financial relationships that could be construed as a potential conflict of interest.
